# Semi-automated selection of cryo-EM particles in RELION-1.3

**DOI:** 10.1016/j.jsb.2014.11.010

**Published:** 2015-02

**Authors:** Sjors H.W. Scheres

**Affiliations:** MRC Laboratory of Molecular Biology, Cambridge Biomedical Campus, Francis Crick Avenue, Cambridge CB2 0QH, UK

**Keywords:** Electron cryo-microscopy, Single-particle analysis, Automated particle picking

## Abstract

The selection of particles suitable for high-resolution cryo-EM structure determination from noisy micrographs may represent a tedious and time-consuming step. Here, a semi-automated particle selection procedure is presented that has been implemented within the open-source software RELION. At the heart of the procedure lies a fully CTF-corrected template-based picking algorithm, which is supplemented by a fast sorting algorithm and reference-free 2D class averaging to remove false positives. With only limited user-interaction, the proposed procedure yields results that are comparable to manual particle selection. Together with an improved graphical user interface, these developments further contribute to turning RELION from a stand-alone refinement program into a convenient image processing pipeline for the entire single-particle approach.

## 1 Introduction

Recent advances in electron cryo-microscopy (cryo-EM) single-particle analysis have made it possible to obtain near-atomic resolution structures for a much wider range of specimens and from much fewer particles than before. Previously, cryo-EM maps with sufficient detail to see amino acid side chains could only be obtained for hundreds of thousands asymmetric units of large icosahedral viruses (Grigorieff and Harrison, 2011). However, last year a ribosome reconstruction with details of around 4 Å was reported from 35 thousand (asymmetric) particles (Bai et al., 2013), and a 20S proteasome structure to 3.3 Å was reported from 1.8 million asymmetric units (Li et al., 2013). More recently, a 3.2 Å map for the yeast mitochondrial large ribosomal subunit was reported from 47 thousand particles (Amunts et al., 2014), a 3.4  Å  structure of the F_420_-reducing [NiFe] hydrogenase from 319 thousand asymmetric units (Allegretti et al., 2014), and a 3.4 Å  structure of the TRPV1 ion channel from 142 thousand asymmetric units (Cao et al., 2013).

Two developments play an important role in these advances. The first is the development of direct-electron detectors, which are much more efficient at detecting electrons than conventionally used photographic film or charged-coupled devices (CCDs) (McMullan et al., 2009). The higher detection quantum efficiency (DQE) of the new detectors yield images with much improved signal-to-noise ratios (SNRs). This has a “double effect” on the resolution of 3D reconstructions: not only need one average over fewer particles to obtain a given resolution, but one can also align and classify each particle better, so that reconstructions are blurred to a much smaller extent than before. This then also relates to the second development: that of powerful new image processing algorithms. In particular, unsupervised image classification algorithms may be used to separate projections of distinct 3D structures, so that relatively impure or structurally heterogeneous samples may still lead to high-resolution structure determination, e.g. see (Fernndez et al., 2013; Voorhees et al., 2014). Moreover, as the new detectors are also very fast, one can now record multiple images during irradiation of the sample in the microscope. Since interactions with the incoming electrons cause movement of the sample, movie processing algorithms that correct for these beam-induced movements may further increase resolution (Campbell et al., 2012; Bai et al., 2013; Li et al., 2013). These developments have opened up the possibility to apply high-resolution cryo-EM structure determination to a much wider range of samples than before, which will attract many new researchers to this exciting field.

Together with increased interest in the technique, the call for high-throughput, easy-of-use and automation will also grow. One step in the data processing pipeline of high-resolution structure determination that may take considerable amounts of time and user-input is the selection of particles that are suitable for 3D reconstruction. In the past this process was typically done manually by the researcher, who would sit in front of a computer screen and click on each individual particle. Over the last 15 years, many algorithms to automate this often tedious procedure have been proposed, see (Nicholson and Glaeser, 2001; Zhu et al., 2004) for earlier reviews. More recently, implementations of automated particle picking algorithms were made available in EMAN2 (Tang et al., 2007), SIGNATURE (Chen and Grigorieff, 2007), DOGPICKER (Voss et al., 2009), XMIPP (Sorzano et al., 2009), and ARACHNID (Langlois et al., 2014) among others. These approaches may broadly be divided into two categories: feature-based and template-based approaches. In the feature-based approaches, different characteristics of the particles are expressed in some numerical manner (features) and features calculated from local areas in the micrographs are compared to a set of expected features. In the template-based approaches, images that express the expectation how the particles look like are correlated against the micrographs, often using fast Fourier-transform (FFT) accelerated algorithms (Roseman, 2003, 2004). The distinction between the two types of approaches is not always clear, as sometimes expected features are calculated from template images themselves. In general, template-based approaches introduce a higher degree of prior information into the picking process than feature-based approaches, which may be both an advantage and a disadvantage. The advantage of using more prior information is that it allows to detect weaker signals. However, the high levels of noise in the micrographs also make the picking task extremely prone to reference bias. Thereby, relying heavier on prior information becomes a disadvantage in cases where this information is incorrect.

This paper describes recent developments in the RELION software (Scheres, 2012a,b) that are centred around a new template-based particle picking algorithm. The choice for a template-based approach was motivated by its larger potential to select particles from noisy data. The workflow proposed is a semi-automated one. The researcher manually picks particles from a low number of micrographs; uses reference-free 2D class averaging inside RELION to calculate average images of these particles; performs template-based automated particle picking with those class averages on all micrographs; and then relies on a new sorting algorithm and further 2D class averaging to remove false positive from the data. To facilitate this process an improved graphical user interface (GUI) was also implemented. Whereas RELION was originally proposed as a stand-alone refinement program, these developments continue the evolution of RELION into a software package that provides a convenient pipeline for most of the single particle analysis tasks. Together with an improved movie-processing approach to correct for beam-induced motion in samples of relatively small particles (Scheres, 2014), the developments presented here represent the main improvements in the latest (1.3) release of RELION.

## 2 Approach

### 2.1 Particle picking

**Fig.1:**
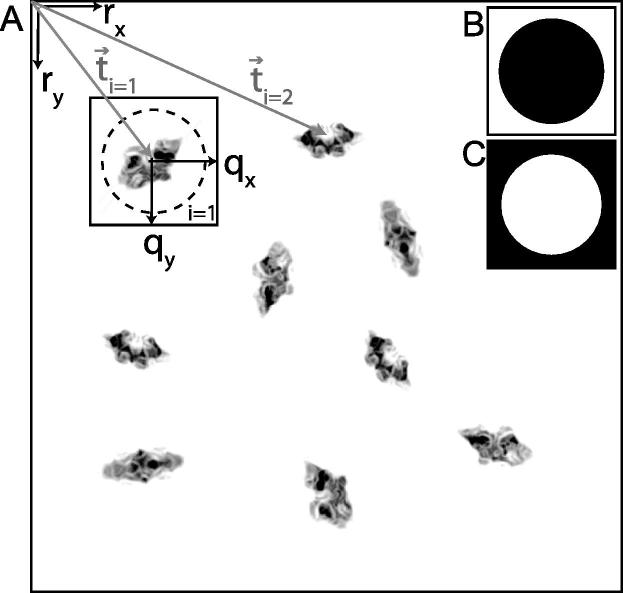
Schematic representation of the data model. (A) Representation of a micrograph, with coordinate vectors $r=(r_{x}\text{,}r_{y})$ inside the micrograph, and coordinate vectors $q=(q_{x}\text{,}q_{y})$ inside each particle image. Vectors $t_{i}=(t_{x}\text{,}t_{y})$ place the *i*th particle inside the micrograph with an unknown in-plane rotation $\phi_{i}$ with respect to a common frame of reference. Inset (B), mask $M_{o}$ which is used for normalisation of the particle images: average and standard deviation of the background pixels are calculated in the white area of this mask. Inset (C), mask $M_{i}$ which is used for the particle sorting algorithm: all statistics on the difference images between each particle and its corresponding template are calculated in the white area of this mask.

The template-based particle picking algorithm proposed employs an additive model with white Gaussian noise in real-space. A micrograph X that contains *N* individual particles *i* at coordinates ${\overset{\to }{t}}_{i}$ in the micrograph is described as follows (also see Fig. 1A):

(1) $$X(\overset{\to }{r})=\mu (\overset{\to }{r})+\sigma (\overset{\to }{r})*\left\{N(\overset{\to }{r})+\overset{N}{\underset{i=1}{\sum }}A_{k_{i}}^{\phi_{i}}(\overset{\to }{r}-{\overset{\to }{t}}_{i})\right\}\text{,}$$

where:

• $X(\overset{\to }{r})$ is the micrograph, i.e. a two-dimensional image that was recorded in the electron microscope, and *r* describes the two-dimensional position in that image.
• $N(\overset{\to }{r})$ is an image of independent (or white) Gaussian noise with mean zero and standard deviation one.
• $\mu (\overset{\to }{r})$ and $\sigma (\overset{\to }{r})$ are position-dependent additive and multiplicative *normalisation* factors that bring the recorded noise levels in the micrograph to mean zero and standard deviation one. Variations in μ and σ with position typically describe experimental variations in ice thickness, electron dose, *etc*.
• $A_{k_{i}}^{\phi_{i}}$ is one of *K* known, two-dimensional *template* images $A_{k}$ with internal positions $\overset{\to }{q}$. Typically, the template images are much smaller than the micrograph (in the summation above, the template image is zero outside the defined box size). Therefore, for any given $\overset{\to }{r}$ and template $A_{k_{i}}$ at position ${\overset{\to }{t}}_{i}$, the internal position will be $\overset{\to }{q}=\overset{\to }{r}-{\overset{\to }{t}}_{i}$. The *K* different template images may describe projections in different directions of the same molecule, or they may describe projections of different molecules; $k_{i}$ describes which of the template images corresponds to the *i*th particle; and $\phi_{i}$ describes the relative in-plane rotation between the particle and that template image.

Given $X(\overset{\to }{r})$ and *K* template images $A_{k}$, the task at hand is to identify all *N* combinations of ${\overset{\to }{t}}_{i}\text{,}\phi_{i}$ and $k_{i}$. Based on positive experiences with maximum-likelihood approaches, e.g. see (Scheres et al., 2007; Scheres, 2012a), the choice was made to implement a probability-based similarity metric for this task. The assumption of Gaussian noise in Eq. (1) naturally leads to a Gaussian similarity measure. Unlike the cross-correlation coefficient, as used for example in the template-based picking program findEM (Roseman, 2003, 2004), the squared difference term inside the Gaussian metric is not invariant to multiplication with or addition of a constant. This means that one needs to account for the varying intensity levels in the recorded micrographs, and one needs to determine the normalisation factors $\mu (\overset{\to }{r})$ and $\sigma (\overset{\to }{r})$ to bring all particles on the same intensity level.

Upon extraction of individual particles from the micrographs, RELION relies on a normalisation procedure that uses a circle (with a user-defined radius *R*, see Fig. 1B) to divide each extracted particle image in a background area (outside the circle) and a particle area (inside the same circle) (Sorzano et al., 2004). By subtracting the average value of the pixels in the background area from the entire particle image, and subsequently dividing the entire image by the standard deviation of the pixels in the background area, noise levels with zero-mean and unity-standard deviation are obtained for all particles, independent of variations in ice thickness, exposure or other uncontrolled experimental factors.

The same normalisation procedure is used inside the implemented particle picking procedure. Using similar concepts as in the fast local correlation algorithm inside findEM (Roseman, 2003, 2004), the values of $\mu (\overset{\to }{r})$ and $\sigma (\overset{\to }{r})$ can be precalculated efficiently for all $\overset{\to }{r}$ using fast Fourier transforms (FFTs):

(2) $$\mu (\overset{\to }{r})=\frac{1}{M_{o}}{\mathrm{FT}}^{-1}\left\{\mathrm{FT}(X)\mathrm{FT}{\left(M_{o}\right)}^{*}\right\}\text{,}$$

(3) $$\sigma (\overset{\to }{r})=\sqrt{\frac{1}{M_{o}}{\mathrm{FT}}^{-1}\left\{\mathrm{FT}(X^{2})\mathrm{FT}{\left(M_{o}\right)}^{*}\right\}-\mu^{2}(\overset{\to }{r})}\text{,}$$

where *FT* and ${\mathrm{FT}}^{-1}$ denote forward and inverse Fourier transform operations, ∗ denotes complex conjugation, and $M_{o}$ is a binary mask as depicted in Fig. 1B with $M_{o}$ white pixels. A second mask $M_{i}$ is the inverse of $M_{o}$ and has $M_{i}$ white pixels (Fig. 1C).

Given the data model in Eq. (1), the probability of observing the micrograph with a particle corresponding to template image $A_{k}$ in orientation ϕ and position $\overset{\to }{t}$ is then given by the multiplication of a Gaussian (with a standard deviation of unity) for each pixel $\overset{\to }{q}$ inside mask $M_{i}$:

(4) $$P\left(X|\overset{\to }{t}\text{,}A_{k}^{\phi }\right)\propto \exp\underset{\overset{\to }{q}\in M_{i}}{\sum }-\frac{1}{2}{\left(\frac{X(\overset{\to }{q}+\overset{\to }{t})-\mu (\overset{\to }{r})}{\sigma (\overset{\to }{r})}-A_{k}^{\phi }(\overset{\to }{q})\right)}^{2}\text{.}$$

The range of values of $P(X|\overset{\to }{t}\text{,}A_{k}^{\phi })$ depends on the number of pixels inside mask $M_{i}$ and the power of the signal in $A_{k}^{\phi }$. In order to define a similarity metric with a pre-defined range, one also calculates the probability of observing the micrograph with only noise at that position, i.e. as for an all-zero template image O:

(5) $$P(X|\overset{\to }{t}\text{,}O)\propto \exp\underset{\overset{\to }{q}\in M_{i}}{\sum }-\frac{1}{2}{\left(\frac{X(\overset{\to }{q}+\overset{\to }{t})-\mu (\overset{\to }{r})}{\sigma (\overset{\to }{r})}\right)}^{2}\text{.}$$

Subsequently, one calculates the ratio of $P(X|\overset{\to }{t}\text{,}A_{k}^{\phi })$ and $P(X|\overset{\to }{t}\text{,}O)$, which will be denoted as $R_{\phi \text{,}k}(\overset{\to }{t})$, using:

(6) $$R_{\phi \text{,}k}(\overset{\to }{t})=\frac{P(X|\overset{\to }{t}\text{,}A_{k}^{\phi })}{P(X|\overset{\to }{t}\text{,}O)}=\exp\underset{\overset{\to }{q}\in M_{i}}{\sum }\frac{X(\overset{\to }{q}+\overset{\to }{t})A_{k}^{\phi }(\overset{\to }{q})}{\sigma (\overset{\to }{r})}-\frac{\mu (\overset{\to }{r})A_{k}^{\phi }(\overset{\to }{q})}{\sigma (\overset{\to }{r})}-\frac{1}{2}{\left(A_{k}^{\phi }(\overset{\to }{q})\right)}^{2}\text{.}$$

If $R_{\phi \text{,}k}(\overset{\to }{t})>1$, the position $\overset{\to }{t}$ is more likely to correspond to a particle $A_{k}^{\phi }$ then to solvent. The expected value for $R_{\phi \text{,}k}(\overset{\to }{t})$ for an image according to the data model in Eq. (1) is calculated as:

(7) $$E\langle R_{k}\rangle =\exp\frac{1}{2M_{i}}\underset{\overset{\to }{q}\in M_{i}}{\sum }{\left(A_{k}\right)}^{2}\text{.}$$

Therefore, one can define a similarity metric that adopts values within a meaningful range by expressing the fraction:

(8) $$S_{\phi \text{,}k}(\overset{\to }{t})=\frac{R_{\phi \text{,}k}(\overset{\to }{t})-1}{E\langle R_{k}\rangle -1}\text{.}$$

The value $R_{\phi \text{,}k}(\overset{\to }{t})-1$ expresses how much more likely the position $\overset{\to }{t}$ is to correspond to a particle $A_{k}^{\phi }$ then to solvent. For perfect signal and white Gaussian noise, this value will be close to $E\langle R_{k}\rangle -1$. In practice, the templates are not perfect and the noise is not white, which results in typical values of $R_{\phi \text{,}k}(\overset{\to }{t})-1$ being smaller than $E\langle R_{k}\rangle -1$. Therefore, useful threshold values for peak searching in $S_{\phi \text{,}k}(\overset{\to }{t})$ often lie within the range (0,1].

The calculation of $S_{\phi \text{,}k}(\overset{\to }{t})$ for all ϕ and *k* can be done efficiently as follows. The calculation of $\sum_{\overset{\to }{q}\in M_{i}}A_{k}^{\phi }$ and $\sum_{\overset{\to }{q}\in M_{i}}{\left(A_{k}^{\phi }\right)}^{2}$ is invariant to $\overset{\to }{t}$ and ϕ, and thus need to be calculated only once for each template. Calculation of the remaining unknown in Eq. (6) for all $\overset{\to }{t}\text{,}k$ and ϕ may again be calculated using FFT-accelerated cross-correlation:

(9) $$\underset{\overset{\to }{q}\in M_{i}}{\sum }X(\overset{\to }{q}+\overset{\to }{t})A_{k}^{\phi }(\overset{\to }{q})={\mathrm{FT}}^{-1}\left\{\mathrm{FT}(X)\mathrm{FT}{\left(A_{k}^{\phi }\right)}^{*}\right\}\text{,}$$

provided that $A_{k}^{\phi }(\overset{\to }{q})=0$ for $\overset{\to }{q}\;\notin \;M_{i}$. For each template image $A_{k}$ and each discretely sampled in-plane rotation ϕ=1,…,Φ, one calculates $S_{\phi \text{,}k}(\overset{\to }{t})$ for all $\overset{\to }{t}$. This involves K×Φ evaluations of Eq. (9), whereas Eqs. (2) and (3) only need to be evaluated once for each micrograph. The user controls Φ through the definition of an angular sampling rate (typically 5 degrees). The FFT libraries used only deal with squared images. Rectangular micrographs are padded with white Gaussian noise to obtain squared images. This is done internally, so that the interaction with the user does not change.

Peak searching is first done independently for all $S_{\phi \text{,}k}(\overset{\to }{t})$. Potential particle positions are only selected for local maxima (where the four neighbouring pixels are smaller than the peak value), and if the peak value is higher than a given user-defined threshold. For each image $S_{\phi \text{,}k}(\overset{\to }{t})$, the peaks are pruned based on a user-defined minimum inter-particle distance. All peaks within this distance from each other are clustered together. Within each cluster, the peak with the highest value of $S_{\phi \text{,}k}(\overset{\to }{t})$ is kept, and remaining peaks within the minimum inter-particle distance of the kept peak are discarded. This is done recursively, such that more than one peak from each cluster may be kept, but all pruned peaks will be at least the minimum inter-particle distance from each other. The pruned peaks for all templates *k* and all in-plane rotations ϕ are then combined, and the combined list of peaks is pruned using the same algorithm. The final result if a list of *N* particle coordinates $\overset{\to }{t_{i}}$, each with a value for the corresponding template $k_{i}$ and in-plane rotation $\phi_{i}$.

Although not explicitly written as such in Eq. (1), the implementation in RELION-1.3 is done in such a way that CTF-corrected template images are provided, and that internally $A_{k}$ is calculated by applying a given Contrast Transfer Function (CTF) of the micrograph to the template images. In this way, a fully CTF-corrected picking algorithm is obtained.

### 2.2 Particle sorting

The new sorting algorithm provides a fast way to identify incorrectly picked particles from the data. For every extracted particle image $P_{i}$, the sorting program subtracts an associated template image $A_{k}$ in its given in-plane orientation ϕ. The resulting difference image is used to calculate an arbitrary number of statistical features. If $P_{i}$ corresponded to a true particle, the difference image should contain only background noise. If $P_{i}$ was incorrectly picked as a particle, then the difference image will contain features that cannot be described by background noise alone. As the sorting depends on the availability of a template image, it may be performed at any stage of the image processing when a 2D-template image has been assigned to each particle: i.e. directly after the auto-picking, or after any 2D or 3D classification or refinement. After 2D classification and auto-picking, $A_{k}$ will correspond to one of the *K* 2D templates used; after 3D classification or refinement, $A_{k}$ will correspond to projections of 3D template(s) in a given direction.

The sorting algorithm itself is similar to the one described in Scheres (2010), where one calculates a *Z*-score for an arbitrary number of features in each particle, and then sorts all particles based on the average of all these *Z*-scores. The features in the sorting algorithm in RELION are all based on the difference image between each particle and its aligned template. In particular, they comprise the mean, standard deviation, skewness and kurtosis of the difference image; as well as the standard deviation between the standard deviations calculated in four quadrants of the difference image. All these values are calculated within mask $M_{i}$ (Fig. 1C).

The resulting average *Z*-scores may then be used for displaying the particles in a sorted manner. Particles with a high average *Z*-score are often high-contrast false positives such as ice, protein aggregates, carbon edges or pieces of junk, e.g. Fig. 3C. Therefore, visual inspection of the particles on the high-end of the sorted average *Z*-scores may be a more efficient way of getting rid of bad particles than inspecting the entire data set.

### 2.3 Improved GUI and proposed workflow

**Fig.2:**
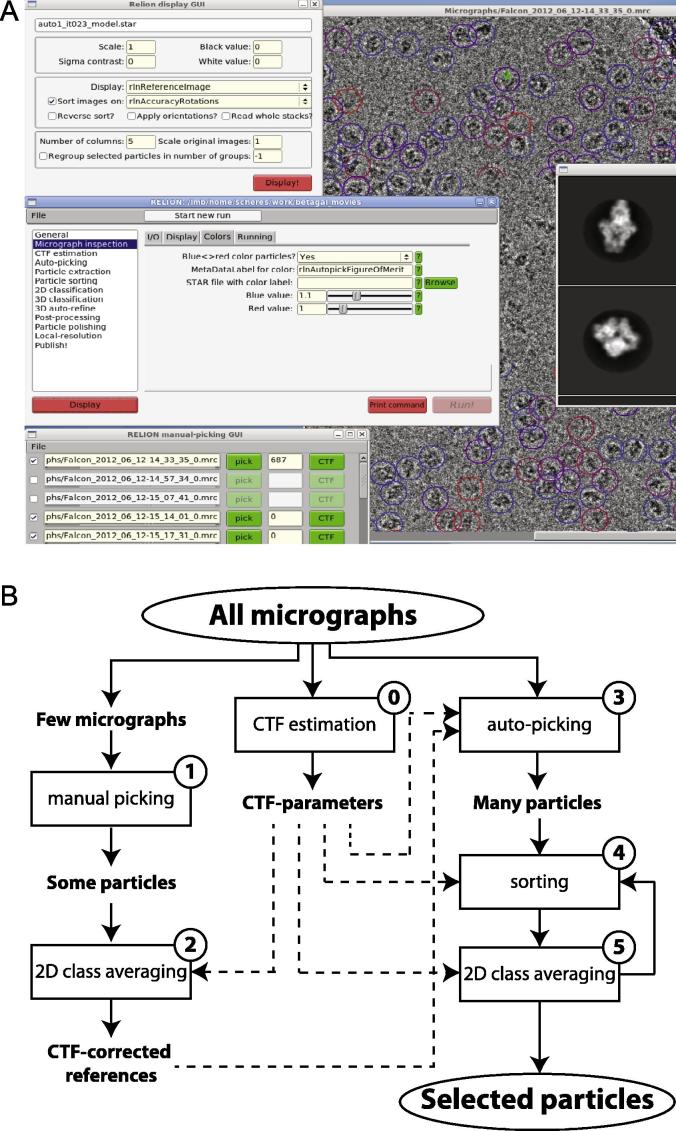
Improved GUI and workflow. (A) Screenshot of the new GUI in RELION-1.3. (B) Proposed workflow for semi-automated particle selection in RELION-1.3. After CTFs have been estimated for all micrographs, the particle selection procedure consists of five steps (numbered 1–5), as explained in more detail in Section 4.

Apart from the new algorithms described above, RELION-1.3 also features an improved graphical user-interface (GUI). Whereas previous releases still relied on user input from the command-line, e.g. using linux-based “awk” commands to select particles from specific classes, all of the functionalities described in this paper may now be performed from the GUI. To that purpose, a new image display program has been developed that reads the RELION-specific metadata files in STAR format (Hall, 1991). This provides the user with a convenient tool to manually inspect micrographs and pick particles, display extracted particles in a given order, write out STAR files with particles from a selection of classes, or display aligned particles from any given class. Fig. 2 shows a screen shot of the new interface, as well as a schematic of the proposed semi-automated particle selection procedure that links together the auto-picking, sorting and reference-free 2D class averaging algorithms (Scheres, 2012b).

## 3 Experimental procedures

The particle selection procedure outlined above was tested on two previously published data sets. Firstly, it was tested on the keyhole limpet hemocyanin (KLH) data set that was used as a benchmark for testing particle picking algorithms in the so-called particle selection “bakeoff” (Zhu et al., 2004). This data set consists of 82 defocus pairs of micrographs at 2.2 Å/pixel that were acquired on a Philips CM200 microscope at 120 kV using a 2k×2k Tietz CCD camera and the Leginon system (Suloway et al., 2005). The first micrograph of each pair was recorded near to focus (NTF: 1 μm); the second one was recorded far from focus (FFF: 3 μm). The accumulated dose on each micrograph was approximately 10 electrons per Å^2^. In the bakeoff, particles were selected either manually or (semi-) automatically using different computer programs, and all results were compared to each other. In the results described below, the particles selected by the algorithms in RELION are compared to the manually selected coordinates by Mouche, who only picked side views without any additional overlapping densities.

Secondly, we also applied the new particle selection procedure to a previously described data set on β-galactosidase (Scheres and Chen, 2012; Chen et al., 2013; Vinothkumar et al., 2014). These images, with a calibrated pixel size of 1.77 Å, were recorded manually on an FEI Falcon-II direct-electron detector using an FEI Polara microscope that was operated at 300 kV. An in-house developed system was used to intercept the recorded movies at a frame rate of 16 frames per second. Although in the original data set the exposure times varied from 1.5 s (24 frames) to 5 s (84 frames), only the first 24 frames of all movies were taken into account to calculate new average micrographs with an accumulated dose of 24 electrons per Å^2^. In this case, particles selected in RELION were compared to a manually picked data set by Richard Henderson. Although he originally picked particles in 89 micrographs, for only 84 of those the movies were available, and only those 84 micrographs were used in this paper.

All calculations were performed on Dell M620 computing nodes of twelve 2.8 GHz Xeon cores and 48 Gb of RAM each.

## 4 Results

### 4.1 Particle selection for the standard KLH data set

The recommended semi-automated particle selection procedure in RELION-1.3 consists of at least five steps (Fig. 2B), each of which requires intervention by the user.

**Fig.3:**
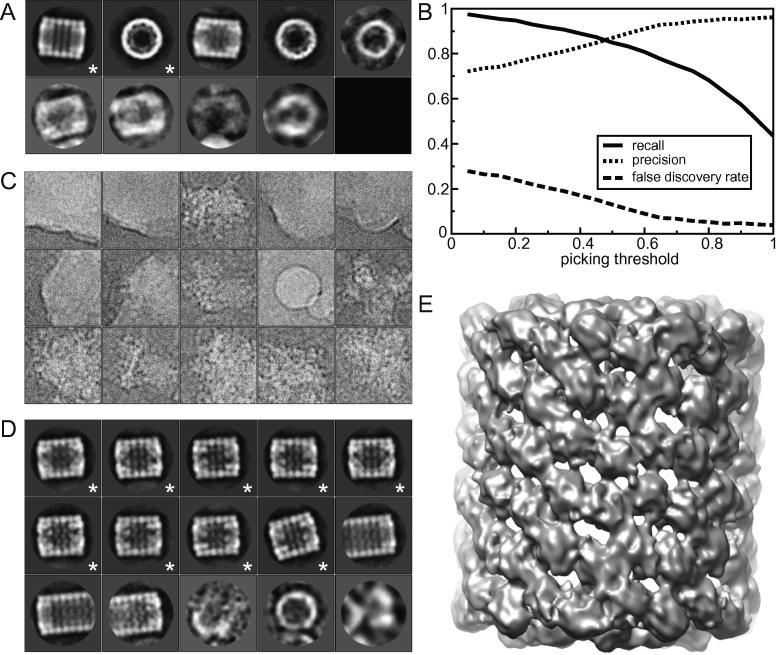
Particle selection for the KLH data. (A) The ten reference-free class averages (ordered from larger to smaller classes) that were calculated from the manually selected particles. The two classes indicated with an asterisk were selected as templates for the auto-picking. (B) Curves of precision, recall and false discovery rate against the pick threshold. A picking threshold of 0.3 was chosen. (C) The 15 particles with the highest average *Z*-scores after sorting. (D) The 15 largest classes (ordered from larger to smaller) after 2D class averaging of the auto-picked particles. Particles assigned to the classes indicated with an asterisk were selected for subsequent 3D refinement. (E) 3D map after refinement of the semi-automatically selected particles from the combined near-to-focus (NTF) and far-from-focus (FFF) KLH data sets.

In the first two steps, the user manually selects particles from a subset of the micrographs and uses these particles to calculate reference-free 2D class averages. The number of particles necessary to calculate suitable templates ultimately depends on the SNR of the data, but 50–100 cryo-EM particles per template appears to be a useful guideline. In this case, from the first ten FFF micrographs, 264 particles were selected manually in step one. At this point, both top and side views were included. In the second step, reference-free 2D class averaging with 10 classes yielded two classes that were much larger than the others, representing a side view and a top view (Fig. 3A). These two class averages were selected to be used as templates for the particle picking algorithm. To prevent model bias, or “Einstein-from-noise” artefacts (also see Section 5), these templates were low-pass filtered to (strictly) 20 Å.

In the third step, one performs the actual automated particle picking. At this point, there are two parameters to be optimised: the picking threshold, with higher values resulting in smaller, cleaner data sets; and the minimum inter-particle distance. The user-controlled angular sampling rate was kept fixed at 5 degrees for all calculations in this paper. Depending on the shape of the templates and the SNR in the micrographs, useful values of the threshold may vary from one data set to another. Fig. 3B shows how the recall, precision and false discovery rate of the auto-picking algorithm vary with the picking threshold. If TP is the number of particles that are both selected by RELION and Mouche, FP is the number of particles that is selected by RELION but not by Mouche, and FN is the number of particles that is selected by Mouche but not by RELION, then recall =TP/(FN+TP), precision =TP/(FP+TP), and false discovery rate (FDR) =FP/(FP+TP), also see (Langlois and Frank, 2011). The minimum inter-particle distance often requires less optimisation, as values of around 60–90% of the particle diameter have been found to be useful in many cases. To accelerate the testing of different values for the picking threshold and the minimum inter-particle distance, the auto-picking program allows one to write out intermediate images with the optimal values (over all ϕ) of $S_{\phi \text{,}k}(\overset{\to }{t})$ for each template *k*. These so-called FOM maps are micrograph-sized images and writing many of them to disk may quickly become a bottle neck. Therefore, the parallel version of this program is disabled when writing out FOM maps, and it is recommended to write FOM maps only for a few representative micrographs of the data set. Once the FOM maps have been written to disk, reading them back in again and picking peaks with different values of the picking threshold and the minimum inter-particle distance may be done in seconds. This allows one to optimise these values for the chosen representative micrographs. The optimised values may then be used to pick particles in the entire data set. In this run one no longer writes out FOM maps, and the program may be run in parallel to speed up the calculations. Two micrographs were selected for the parameter optimisation, which resulted in a picking threshold of 0.3 and a minimum inter-particle distance of 300 Å (which is approximately two-thirds of the diameter of mask $M_{i}$). Auto-picking of all 82 micrographs was done in 5 min using 41 cores in parallel, i.e. taking approximately 2.5 min per micrograph on each core. The RAM requirements for the auto-picking were approximately 400 Mb. Since the manually selected data set only contained side views, at this point all particles that were picked as a top view were discarded.

In the fourth step, one sorts the autopicked particles using the algorithm described in Section 2.2, one displays the particles sorted on the calculated average *Z*-scores, and one manually discards bad particles with the highest average *Z*-scores. Using a single core, the average *Z*-score calculation took less than a minute for the 1195 selected KLH side views. Manual inspection of the approximately 80 particles with the highest average *Z*-scores led to the removal of 44 particles, and was done in less than 2 min. Fig. 3C shows the 15 particles with the highest average *Z*-score as an example of what type of particles get discarded at this stage.

Finally, in the fifth step, the remaining particles are subjected to reference-free 2D class averaging, and manual inspection of the resulting classes is used to discard those particles that do not average into good classes. In this case, the 2D class averaging with 25 classes was performed in 7 min using 36 cores in parallel, and 1048 particles were selected from 9 good classes (Fig. 3D). Depending on the sample, one may repeat the 2D class averaging step several times, and/or also perform 3D classification to further enrich the data set. Also, after any of these steps, one may re-calculate the average *Z*-scores with the improved templates. For this paper, such additional classifications or sortings were not performed.

To further demonstrate the potential of the particle selection procedure for closer to focus images, it was also applied to the 82 NTF micrographs. The comparison of the selected particles after each step for both the FFF and the NTF micrographs with the Mouche coordinates is shown in Table 1. These results suggest that in some cases collecting defocus pairs may actually not be necessary when semi-automatically selecting particles in RELION. The combined 2112 particles from both the FFF and the NTF micrographs were used directly in a 3D refinement, using the preliminary 3D reconstruction that is distributed with these data as an initial model. This refinement yielded a reconstruction with a resolution of 11 Å  according to the gold-standard FSC = 0.143 criterion (Scheres and Chen, 2012) (Fig. 3E).

### 4.2 Particle selection for the β-galactosidase data set

**Fig.4:**
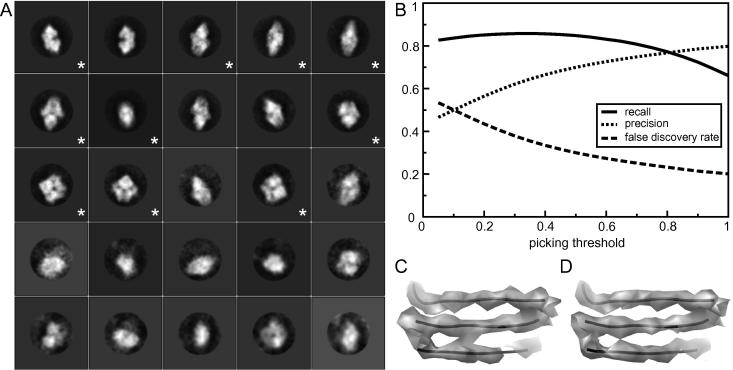
Particle selection for the β-galactosidase data. (A) The 25 class averages that were calculated from the manually selected particles (ordered from larger to smaller class). The 10 class averages indicated with an asterisk were selected as templates for the auto-picking. (B) Curves of precision, recall and false discovery rate against the pick threshold. A picking threshold of 0.4 was chosen. (C) Map obtained after 3D refinement with the manually picked particles. (D) Map obtained after 3D refinement with the semi-automatically selected particles.

The same five-step particle selection procedure was also applied to the β-galactosidase data set. Manual picking in 5 micrographs yielded 2555 particles, which were used for a first 2D classification into 25 classes, and 10 of the resulting class averages were used as templates in the autopicking procedure (Fig. 4A). The picking threshold and the minimum inter-particle distance were set to 0.4 and 130 Å, respectively. Fig. 4B shows the performance of the picking algorithm for different thresholds. Auto-picking all 84 micrographs took approximately 1 h on 43 cores (i.e. taking approximately half an hour per micrograph on a single core), and took approximately 1.5 Gb of RAM.

In this case, the auto-picking algorithm was observed to give obviously false positives for some micrographs that showed high-variance artifacts like edges of the carbon holes or dust particles. Most probably this is caused by low values for $P(X|\overset{\to }{t}\text{,}O)$ in the denominator of $R_{\phi \text{,}k}(\overset{\to }{t})$. Therefore, in experimental applications it may be beneficial to manually supervise the auto-picking results by deleting obviously false positives in the micrographs. The new display program makes this task relatively straightforward. However, in what follows such intervention was not performed in order to better reflect the recall and false discovery rate for the proposed algorithms with minimal user-interaction.

Sorting of the 52,495 particles that were picked automatically took 2 min on 8 cores. After visual inspection, the 4185 particles with the highest average *Z*-scores were discarded. Many of these were corresponding to the obviously false positives in the high-variance regions of the micrographs. The remaining particles were subjected to 2D classification with 200 classes, which took approximately 16 h on 64 cores. Good classes showed white particles with protein-like details on a black background, whereas many bad classes showed low-resolution blobs or images with many features in the background. From a total of 39 selected classes a final number of 42,755 particles were selected.

Table 2 shows the comparison between the particle sets at each of these steps and a manually selected data set of 40,863 particles by Richard Henderson. To further compare the two particle sets, a 3D refinement with the semi-automatically selected particle set was compared with an identical 3D refinement of the manually selected particles. In both cases a 60 Å low-pass filtered crystal structure of β-galactosidase (PDB PDB3I3I3E) (Dugdale et al., 2010) was used as an initial model. These refinements led to a resolution of 4.2 Å for both data sets, and the density map of the semi-automatically selected particles appears to be at least as good as the manual one (*cf* Fig. 4C and D).

## 5 Discussion

The new semi-automated particle picking and sorting algorithms, in combination with the selection of good classes after reference-free 2D class averaging lead to relatively high recalls and low false discovery rates when compared to alternative approaches in the original bakeoff study (Zhu et al., 2004), even when using the near-to-focus KLH micrographs. However, in the bake-off only particle picking approaches were compared, whereas in the approach described here lower thresholds may be used to avoid false negatives at the picking stage, and the sorting and 2D-classification algorithms may be used to remove false positives. For the β-galactosidase data set, refinement of a data set that was selected semi-automatically led to a map that was as least as good as a map obtained from manually picked particles. Therefore, the procedures proposed here may be an attractive alternative to the tedious process of manual particle selection. To facilitate future comparison with other automated picking approaches, the β-galactosidase micrographs and the manually selected coordinates from Richard Henderson were uploaded to the EMPIAR data base at the EMDB (entry EMPIAR-10017).

**Fig.5:**
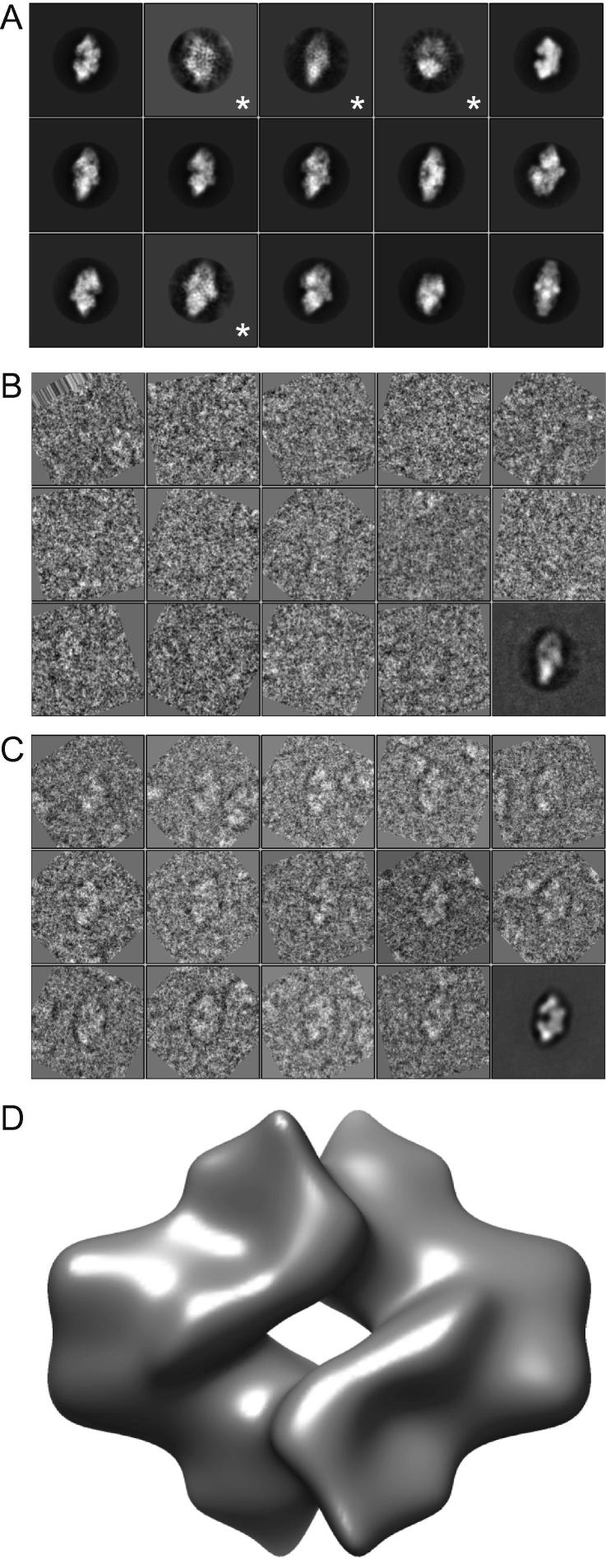
The “Einstein-from-noise” pitfall. (A) Class averages for the 15 largest classes (ordered from larger to smaller) after sorting and 2D class averaging of the auto-picked particles that were picked with a threshold of 0.1. Class averages indicated with an asteriks were identified as artificial classes caused by template bias (see Section 5). (B) Examples of particle images assigned to one of the artificial classes: the third class in A. No clear particles are visible. The lower-right image shows the average of all assigned particles in this class without any CTF correction. (C) Examples of particle images assigned to a good class: the first class in A. Particles are clearly visible. The lower-right image shows the average of all assigned particles in this class without any CTF correction. (D) 3D map obtained from 17,082 particles that were assigned to artificial classes.

However, template-based particle picking does come with a potentially dangerous pitfall. As was pointed out recently in a series of comments on a controversial cryo-EM structure of the HIV-1 envelope glycoprotein trimer (Henderson, 2013; van Heel, 2013), using templates to select particles from noisy micrographs may be subject to strong template bias. This was termed “Einstein-from-noise”, in reference to the classical experiment where pure-noise images are aligned to a picture of Einstein in order to reproduce the Einstein image from averaging over noise only, see also (Shatsky et al., 2009). The template-based picking algorithm in RELION does not form an exception to this general problem. To illustrate this, the auto-picking algorithm was re-run on all β-galactosidase micrographs, but this time with a much lower threshold of 0.1. This led to 70,942 particles being picked, of which 62,230 were selected after sorting. Reference-free 2D class averaging with these particles revealed several artificial, “Einstein-from-noise” classes (indicated with an asterisk in Fig. 5A). Whereas good classes show high-resolution protein-like features, the artificial classes show merely low-resolution ghosts of the templates with superimposed high-resolution noise. Another noticeable difference between these classes is the angular accuracy that RELION estimates (Scheres, 2012b): for true classes this accuracy is often better than for artificial classes. Analysis of the individual particles that were assigned to the artificial classes shows that they are mostly empty particles (Fig. 5B). Moreover, averaging of these particles (without CTF-correction or masking) shows a black circle around the ghost image of the template. This black circle is the ghost image of the circular mask around the template image, which had slightly negative, i.e. black, values in the background. Particles assigned to good classes are clearly visible in individual images, and averaging over these does not show the black circle (Fig. 5C), which should not be mistaken for the typical black “aura” around an average that has not been CTF-corrected.

The extent of bias in template-based particle picking is not to be under-estimated. For the reference-free class averages shown in Fig. 5A, the second, third and fourth most populated 2D classes were identified as artificial classes. In order to be able to distinguish these false classes from the true ones, it is highly recommended to low-pass filter the templates used for auto-picking. For both the KLH and the β-galactosidase data sets, the auto-picking templates were filtered using a filter that strictly drops to zero beyond 20 Å. As selection of individual particles from noisy micrographs is mainly driven by relatively low frequencies, low-pass filtering of the templates is not expected to have a large impact on the results. But, the artificial class averages will not contain any features beyond that resolution, and true classes may then easily be distinguished from false ones if they contain useful features to higher resolution. Even if artificial classes were to be incorrectly included at this point, a subsequent gold-standard 3D refinement with only such particles would not be expected to reach resolutions beyond the low-pass filter used. To illustrate this, 17,082 particles that were assigned to artificial classes were subjected to 3D refinement, which yielded a featureless reconstruction at a reported resolution of 29 Å (Fig. 5D). It is important to note that the opposite is certainly not true. If the template images used in the auto-picking were not low-pass filtered, then model bias in the picked particles might still lead to spuriously high FSC values, regardless whether the FSC was calculated using gold-standard procedures, or whether some sort of reference-free 2D class averaging had been performed in between (van Heel, 2013). Therefore, one should not trust any reconstruction with a resolution that does not extend beyond the resolution of the templates that were used for the particle-picking.

Taken together, the new algorithms presented here in combination with an improved graphical-user interface provide a streamlined processing workflow for single-particle analysis in RELION. With the notable exception of initial model generation, all steps of a single-particle structure determination may now be performed from the RELION-1.3 interface: starting with initial micrograph inspection and ending at the generation of a final map that is suitable for atomic model building and refinement. As in previous releases, RELION-1.3 relies on CTFFIND3 (Mindell and Grigorieff, 2003) for CTF determination, and the new release employs a wrapper to RESMAP (Kucukelbir et al., 2014) to calculate local resolution variations in the final reconstruction. RELION-1.3 is open-source software and may be downloaded from http://www2.mrc-lmb.cam.ac.uk/relion. The semi-automated particle selection workflow presented here has already been useful in our own research on a 3.2 Å  structure of the *Plasmodium falciparum* ribosome (Wong et al., 2014) and a 4.5 Å  structure of the human γ-secretase complex (Lu et al., 2014). Hopefully, it will also contribute positively to the research of others who wish to use cryo-EM structure determination.

## Figures and Tables

**Table 1:** Number of selected particles for each particle set, and the recall and false discovery rate (FDR) at the different steps in the RELION procedure for the far-from focus (FFF) and near-to-focus (NTF) data sets.

Data set	Nr. selected	Recall	FDR
Mouche	1042	–	–
FFF step 3	1195	0.92	0.20
FFF step 4	1151	0.91	0.18
FFF step 5	1048	0.92	0.10

NTF step 3	1261	0.91	0.25
NTF step 4	1173	0.91	0.19
NTF step 5	1064	0.90	0.12

**Table 2:** Number of selected particles for each particle set, and the recall and false discovery rate (FDR) at the different steps in the RELION procedure for the β-galactosidase data set.

Data set	Nr. selected	Recall	FDR	
Henderson	40,863	–	–	
Step 3	52,495	0.85	0.34	
Step 4	48,310	0.81	0.31	
Step 5	42,755	0.79	0.25	
